# From crop left-overs to nutrient resource: growth-stimulating potential of biochar in nutrient solutions for wheat soilless cultivation systems

**DOI:** 10.3389/fpls.2024.1414212

**Published:** 2024-09-05

**Authors:** Kris Kunnen, Md Muntasir Ali, Amine Lataf, May Van Hees, Robin Nauts, Nele Horemans, Dries Vandamme, Ann Cuypers

**Affiliations:** ^1^ Environmental Biology, Centre for Environmental Sciences (CMK), Hasselt University, Diepenbeek, Belgium; ^2^ Biosphere Impact Studies, Belgian Nuclear Research Centre (SCK CEN), Mol, Belgium; ^3^ Analytical and Circular Chemistry, Institute for Materials Research (imo-imomec), Hasselt University, Diepenbeek, Belgium

**Keywords:** biochar, wheat, hydroponics and soilless culture, plant growth, *Arabidopsis thaliana*, circular bioeconomy

## Abstract

To reach the estimated food demands for 2050 in decreasingly suiting climates, current agricultural techniques have to be complemented by sustainably intensified practices. The current study repurposed wheat crop residues into biochar, and investigated its potential in different plant cultivation systems, including a hydroponic cultivation of wheat. Biochars resulting from varying pyrolysis parameters including feedstock composition (straw and chaff) and temperature (450°C and 600°C), were tested using a fast plant screening method. Biochar WBC450, produced from a combination of chaff and straw at 450°C, was selected for further plant experiments, and used in a static leaching experiment in the *Arabidopsis thaliana* cultivation medium. Increased pH and EC were observed, together with an increase of most macronutrient (K, Mg, P, S) and a decrease of most micronutrient (Fe, Mn, Zn) concentrations. Considering plant growth, application of biochar resulted in concentration-dependent effects in both tested plant species (*A. thaliana* and wheat). It improved the vegetative yield across all tested cultivation systems. Increases in K and S, and concentration-dependent decreases in Fe and Na content in wheatgrass were observed. Biochar influenced the reproduction of hydroponically cultivated wheat by increasing the number of spikes and the number of seeds per spike. The antioxidative capacity of wheat grass, and the seed sugar and starch contents remained unaffected by biochar application. This study contributes to innovation in soilless cultivation approaches of staple crops, within the framework of closing waste loops for a circular bioeconomy.

## 1 Introduction

The world population is expected to rise above 9 billion by 2050, accompanied by an increase in food demand of at least 70% (Godfray et al., 2010; Asseng et al., 2020). Simply upscaling current industrial agricultural practices to meet the increase in food demand is not considered sustainable: conventional agricultural systems consume extensive amounts of fresh water and depend largely on pesticides and fertilizers, hereby negatively affecting the environment (Weber, 2017). Additionally, producers are put under pressure by decreasing arable land, availability of water and nutrient resources, rising urbanization and climate change (Gruda, 2019; Tseng et al., 2019).

New approaches for crop production, based on principles of sustainable intensification, are needed to complement current cultivation practices (Godfray et al., 2010; Zhou et al., 2021). Examples include food production in greenhouses (Zhou et al., 2021), or strategies like urban farming. The field of urban farming encompasses challenges of urban population growth, food quality, farmland shortages, food miles, greenhouse gas issues, organic waste management and sustainable fertilizers, as well as the support of local economy (Al-Kodmany, 2018; O'Sullivan et al., 2019). For example, a vertical farm in particular has the benefit of increasing crop yields per land area used. Additionally, as with other strategies in urban farming, vertical farms can be more efficient in terms of water use efficiency and fertilizer use (Arabzadeh et al., 2023). Applying circularity principles in urban farming can increase its sustainability, for example by implementing a closed irrigation system. A study by Rufí-Salís et al. (2020) indicated that a closed system opposed to a linear cultivation system of common bean (*Phaseolus vulgaris* L.) saved 40% water and at least 35% nutrients. They also described that using a closed irrigation system significantly reduced the effect on eutrophication, induced by discarding fertilizers into the environment (Rufí-Salís et al., 2020).

In addition to adjusting technical aspects of plant cultivation, attention should be drawn to the sustainable management of residual, non-edible parts of crops (Tseng et al., 2019). Repurposing crop residues falls within the framework of circular bioeconomy, where the conversion of biological waste streams into value-added products is considered an innovative research-based approach (Nematian et al., 2021; Santolini et al., 2021). In this way, circular bioeconomy contributes to sustainable intensification of alternative agricultural practices.

Biochar production from residual crop waste is a promising technology that fits the development of circular economy in the agricultural sector (Jindo et al., 2020). As a by-product of the pyrolysis of biomass, biochar is regarded as a negative emission technology. During pyrolysis, 63-82% of the initial carbon is sequestered instead of being released into the atmosphere (Woolf et al., 2021). Biochar can affect the physicochemical characteristics or microbial activity of the soil or substrate it is amended to. These influences on soil or substrate properties can be attributed to the physicochemical characteristics of biochar, which in their turn are influenced by pyrolysis production parameters (Lataf et al., 2022). Resulting effects of biochar on plant growth, both in soil (Joseph et al., 2021) and in substrate cultivation systems (Huang and Gu, 2019), have been extensively researched and reviewed. In soils, biochar induces an average increase in crop yield of 10-42%. This outcome on plant growth is described as largely dependent on the interplay between pyrolysis parameters (such as temperatures), biochar application rate, as well as the application context (e.g. initial soil characteristics) (Joseph et al., 2021). Additionally, a recent systematic review by Bekchanova and colleagues indicated a 20% increase in crop yield when biochar was amended to sandy soils (Bekchanova et al., 2024). The review of Huang and Gu (2019) on biochar amendment in substrate-based cultivation, found that in 77% of their reviewed studies biochar application induced a growth promoting response. However, they argued that non-herbaceous plants were underrepresented in their reviewed research articles (Huang and Gu, 2019). Compared to soil- and substrate-based cultivations, few studies investigated the implementation of biochar in hydroponic cultivation of staple food crops.

This study describes research results from the SpaceBakery project, a Flemish innovation project coordinated by Flanders’ FOOD, bringing together industrial and academic partners for studying the potential of hydroponically cultivating wheat and baking bread from harvested grains in a completely closed loop. Challenges in this project overlapped with the above-mentioned strategies to sustainably intensify urban agriculture, as some focus lay on repurposing wheat crop residues. This is considered a challenge for closed environment cultivation systems (Nelson et al., 2008), rendering it an interesting case study for the concept of circular bioeconomy. Despite the evident benefits of urban agriculture and vertical farming, high initial investments and energy costs repress the economic viability of staple food crop production, highlighting low-calorie products such as lettuce and herbs as more profitable (Arabzadeh et al., 2023). Asseng et al. (2020), who compared the wheat yield potential in vertical farms, confirmed this doubt on economic viability. Yet, the authors emphasized to continue experimental research into optimizing the production potential, as it could play an essential role in maintaining crop yields in future climates or after unforeseen disturbances in our food systems (Asseng et al., 2020).

Therefore, in this manuscript, the potential of biochar production from wheat residues and its implementation in a soilless cultivation of wheat was investigated. First, biochars resulting from different pyrolysis process parameters (feedstock composition, temperature) were compared. Then, the effects of biochar on development and growth of seedlings (*Arabidopsis thaliana* (L.) Heynh. and *Triticum aestivum* L. (wheat)) were investigated. Lastly, a wheat seed-to-seed experiment on hydroponics was conducted, as a case study for biochar implementation in soilless cultivation systems to fit the circular bioeconomy framework.

## 2 Methods

### 2.1 Experimental workflow

**Figure 1:**
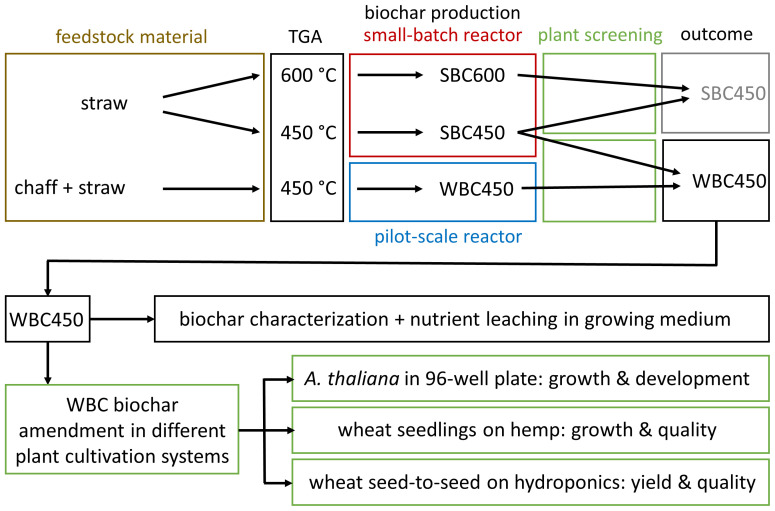
An overview of the experimental workflow. TGA, thermogravimetric analysis; SBC600, straw-based biochar pyrolyzed at 600°C; SBC450, straw-based biochar pyrolyzed at 450°C; WBC450, wheat-based (chaff and straw) biochar pyrolyzed at 450°C.

The description of the experimental workflow (**Figure 1**) provides an overview of the biochar selection and implementation conducted throughout the study. Summarized, biochars resulting from differing pyrolysis parameters (feedstock composition, temperature, pyrolysis reactor), were compared using a fast plant screening method. One favored biochar, WBC450 was then used for further physicochemical characterizations and implementation in different plant cultivation systems.

First, based on a thermogravimetric analysis (TGA) of straw, pyrolysis temperatures (400°C and 600°C) were determined. As the plant screening requires little amounts of biochar, a small-batch reactor (pyrolysis reactors are described below) was used to produce biochar from low amounts (40 grams) of wheat straw. This resulted in straw-based biochar, produced at 450°C and 600°C (SBC450 and SBC600). In a second screening experiment, biochar from combined wheat chaff and straw, produced at 450°C in a bigger pilot-scale reactor (WBC450) was compared to SBC450. This comparison was made for two reasons. First, the TGA analysis pointed out that wheat straw and chaff had a similar degradation pattern, except for a differing ash content (described in results). Secondly, Haeldermans et al. (2023) pointed out that the pilot-scale reactor, able to process larger amounts of feedstock ( ± 1.5 kg), is suitable for upscaling production compared to biochar produced with the small-batch reactor (Haeldermans et al., 2023). Continuing with WBC450 seemed most interesting for several reasons. The choice regarded the plant screening outcome and the energy consumption for its production. Also, it utilized the majority of the crop waste, supporting the circular bioeconomy concept. Therefore, WBC450 was further characterized and implemented in different plant cultivation systems. These included the 96-well plate cultivation system, the cultivation of wheat seedlings (or wheatgrass) grown on hemp and a wheat seed-to-seed experiment on hydroponics.

### 2.2 Wheat biomass treatment, biochar production and treatment

To reduce the moisture content of the biomass to below 20%, wheat straw and chaff were either air dried or dried in an oven at 45°C for at least 48 hours. The amount of chaff in the total feedstock for WBC450 was 18.5%, based on the total dry weights of wheat straw and chaff in other cultivation experiments of the SpaceBakery project. Roots from wheat plant were not included in the feedstock, as they could not be untangled from the basaltic rocks used as the cultivation substrate.

Straw-based biochar was produced using conventional pyrolysis in a home-built small-batch reactor, described in Vanreppelen et al. (2014), following a procedure described in Vercruysse et al. (2021). Feedstock input was 40 grams for each run. The pyrolysis heating rate was set to 20°C/min, up until the set temperatures of 450°C (SBC450) and 600°C (SBC600) under a nitrogen flow rate of 70 mL/min. An isothermal period of 30 minutes was maintained after the set temperature was reached (Vanreppelen et al., 2014; Vercruysse et al., 2021).

With more feedstock available (± 1.5 kg for one run), WBC450 was produced in a modified rotary kiln pilot-scale reactor, described in Haeldermans et al. (2019), following a procedure described in Lataf et al. (2022). The reactor consisted of a screw feeder (350-400 g/h), followed by a kiln, indirectly heated to 450°C. The inclination and rotational speed were adjusted to have a mean particle residence time of approximately 15 minutes (Haeldermans et al., 2019; Lataf et al., 2022).

### 2.3 Biomass and biochar properties

#### 2.3.1 Determination of pyrolysis temperature: proximate analysis of feedstock

A thermogravimetric analysis of wheat straw and chaff was carried out using a Q500 apparatus (TA instruments, New Castle, USA), following a temperature program described in Vercruysse et al. (2021) (Vercruysse et al., 2021): under a dynamic nitrogen atmosphere (90 mL/min), an initial heating period of 20°C/min was applied from room temperature to 600°C. An isothermal period of 3 minutes was set at 600°C before switching the atmosphere to oxygen (90 mL/min). Subsequently, another heating ramp (20°C/min) was applied until the final temperature of 900°C was reached. Sample sizes were 9.94 and 5.8 mg for chaff and straw respectively.

#### 2.3.2 Ultimate analysis

Using a Thermo Electron Flash EA1112 elemental analyzer (ThermoFisher Scientific, Waltham, USA), total carbon, hydrogen, nitrogen and sulfur content of biochar WBC450 were determined. Calibration was performed using BBOT ((2,5-bis (5-tert-butyl-benzoxazol-2-yl) thio-phene) (pure, ThermoScientific)). Total oxygen content was calculated by difference (O (wt%) = 100% - C (wt%) – N (wt%) – H (wt%) – S (wt%) – ash (wt%)). Ash content of wheat chaff, straw and WBC450 biochar was determined using method ASTM D2866 – 94.

### 2.4 Mineral leaching experiment

To determine the effect of WBC450 on the mineral composition of the ¼ MS medium, used in the fast plant screening method (described below), excluding possible influences of plants themselves, a static leaching experiment was conducted. The ¼ MS cultivation medium was chosen for this objective, as it is the least influenced by other factors such as replenishing water or other solutions, or variability in hemp composition in other cultivation systems. This way, differences can be attributed to biochar with more certainty. Biochar was amended to 20 mL ¼ MS medium, in concentrations of 0 mg/ml, 0.25 mg/ml, 0.5 mg/ml and 1 mg/ml. These biochar concentrations were chosen as they are the same as the ones used in the plant experiment considering the flow cytometric analysis of nuclear ploidy levels, described below. These two experiments were performed at the same time. The concentrations are also similar to the ones used in the plant-based comparison of different biochars, with the addition of a lower concentration of 0.25 mg/ml, in order to explore the concentration-dependent effect of biochar. After 7 days of statically remaining in the climate chamber, samples were filtered through a 0.2 µm filter and acidified (HNO_3_) in function of inductively coupled plasma – atom emission spectroscopy (ICP-AES) (Vercruysse et al., 2024), using distilled water as a blank. Electrical conductivity (EC) and pH were determined in filtered, non-acidified volumes.

### 2.5 Biochar screening and effect on plant development

#### 2.5.1 Plant phenotypic screening of biochar

To compare biochars that result from different production parameters (feedstock composition, reactor or temperature), a quick plant development screening was conducted, using *Arabidopsis thaliana*. Seeds of wild-type (WT) *A. thaliana* (ecotype: Columbia) were surface sterilized using 70% ethanol. After 2-3 nights in the dark at 4°C for stratification, seeds were sown in 96-well plates containing autoclaved ¼ Murashige-Skoog (MS) medium supplemented with sucrose (5 g/l) in distilled water. Biochar was amended in medium using concentrations ranging from 0 mg/ml to 2 mg/ml. Plates were kept in a climate-controlled chamber on a 12-hour photoperiod, 22°C/18°C day/night temperature cycle, 65% humidity and a photosynthetic active radiation (PAR) of 170 µmol m^-2^ s^-1^ emitted as blue, red and far red light from Phillips Green-Power LED-modules (Hendrix et al., 2018). At 7 DAS, root length was measured. At 10 or 11 days after sowing (10 DAS, 11 DAS), root length and fresh weight were determined. Values of plants exposed to biochar at different concentrations were compared to controls, and plants exposed to similar concentrations of different biochars were compared. This, in order to detect differences in growth-stimulating or -inhibiting effects induced by the compared biochars.

#### 2.5.2 Flow cytometric analysis of nuclear ploidy levels

To determine the effects of biochar (0 mg/ml, 0.25 mg/ml, 0.5 mg/ml, 1 mg/ml) on early plant development, flow cytometric analyses in *A. thaliana* seedlings were conducted using the CyStain® PI Absolute P kit (Sysmex Partec). Plants were cultivated as described above, harvested at 7 DAS, snap frozen in liquid nitrogen and kept in a -80°C freezer until analysis. Four seedlings were pooled for each sample, and chopped in 250 µL extraction buffer with a razor blade. After 15 min of incubation, nuclei were passed through a 50 µm nylon filter (CellTrics®, Sysmex Partec). A staining solution with 1 mL staining buffer, 3 µL RNase A and 6 µL propidium iodide (PI) was added to filtered samples. The mixture was incubated in the dark on ice for at least 1 h. Ploidy levels (% 2C, % 4C, % 8C, % 16C, % 32C, C representing the haploid DNA content) of 8000 nuclei were determined using the CyFlow® Cube 8 flow cytometer (Sysmex Partec). Using a 488 nm laser, PI-stained nuclei were excited. The forward scatter and PI fluorescence intensity (580/30 nm) were measured, and analysed using FCS Express 4 software (*De Novo* Software), resulting in a proxy value and an endoreplication index. The proxy is the number of counted nuclei per µL, measured as a derivation of biochar-induced effects on cell division. The endoreplication index (EI) was calculated using the following formula: ((0 × % 2C) + (1 × % 4C) + (2 × % 8C) + (3 × % 16C) + (4 × % 32C))/100 (Hendrix et al., 2018).

### 2.6 Wheatgrass cultivation

#### 2.6.1 Growth conditions and biochar exposure

The effect of WBC450 on early plant development and quality of wheat seedlings (*Triticum aestivum* cv. Servus), or also described in this manuscript as wheatgrass, was examined in a small box cultivation experiment using a hemp-based substrate. Seeds were surface sterilized in ethanol (70%, 30 s), bleach (2%, 5 min), ethanol (70%, 30 s) and rinsed repeatedly with sterilized distilled water. After sterilization, seeds were incubated at 20°C on wet filter paper in petri dishes for 3 days. The small box set-up consisted of two hemp mats with a total weight of 20 grams. An initial volume of 100 mL distilled water was added to the small box set-up. Biochar (WBC450) was added to 50 mL of the initial water volume, vortexed, and added in between the two hemp layers. On the top layer, the remaining 50 mL water was added and germinating seeds were sown. Biochar concentrations (0 mg/ml, 0.25 mg/ml, 0.5 mg/ml, 0.75 mg/ml) in this small box set-up will be expressed in function of the initial 100 mL water volume. Two-daily, the initial weight of each box was recuperated by adding distilled water to compensate for water loss due to evaporation. Between 7 DAS until the final harvest, boxes were supplemented with 1/10 Hoagland solution (10.1 mM KNO_3_, 300 µM Ca(NO_3_)_2_.4H_2_O, 200 µM NH_4_H_2_PO_4_, 200 µM MgSO_4_.7H_2_O, 1.64 µM FeSO_4_.7H_2_O, 0.81 µM Na_2_-EDTA, 4.63 µM H_3_BO_3_, 0.91 µM MnCl_2_.4H_2_O, 0.03 µM CuSO_4_.5H_2_O, 0.08 µM ZnSO_4_.7H_2_O, 0.06 µM H_2_MoO_4_.H_2_O) instead of water. The same climate chamber and growth conditions were used as described for the plant phenotypic screening of biochar.

#### 2.6.2 Determination of mineral concentration in wheatgrass shoots

To determine the influence of biochar on the mineral concentration of the shoots, wheatgrass was harvested and placed in a -80°C freezer for 1 hour and dried using a lyophilizer. Samples were then fully digested using 69% HNO_3_ and dried in a heat block at 110°C for three times. Finally, samples were dissolved in 2% HCl and submitted for analysis using inductively coupled plasma optical emission spectroscopy (ICP-OES 710, Agilent Technologies).

### 2.7 Wheat seed-to-seed experiment: biochar in hydroponic cultivation of wheat

#### 2.7.1 Hydroponic cultivation of wheat, growth conditions and biochar exposure

Wheat seeds were surface sterilized using the same protocol as described above. Four seeds were sown on top of semi-open cups with basaltic rocks. For each biochar exposure condition, one hydroponics box was filled with six separate cups. These hydroponic boxes were then filled with 2 L of 1/2 Hoagland solution (up concentrated from previous description), and kept at a constant volume of 2 L per box using an automatic pump system. On days of refreshing the nutrient solution, some volume was set aside for pH and EC determination. The nutrient solution in the boxes, as well as in the automatic pump system, were completely replaced every two weeks. Biochar are expressed in function of the first 2 L of nutrient solution (0 mg/ml, 0.075 mg/ml, 0.56 mg/ml, 1.9 mg/ml). To prevent biochar being rinsed away when the nutrient solution is refreshed, biochar was filled into bags made from regenerated cellulose dialysis membrane (Spectra/Por® 6-8 kD, Fisher Scientific). The hydroponic boxes were exposed to an 18-hour photoperiod with a light intensity of 130-170 µmol m^-2^ s^-1^ PAR emitted as blue, deep red and far red light from Philips Green Power LED modules, at an average temperature of 22°C.

#### 2.7.2 Harvest: plant and seed phenotypic analyses

After a cultivation period of 98 days, root length, root fresh and dry weight, shoot length, plant fresh and dry weight, spike number and length, auricle length, seed number and seed fresh weight were determined. As the roots are impossible to disentangle, the longest root length for each cup was determined by measuring from the top of the cup until the tip of the longest root. All root material for each cup was collected, fresh and dry weights were determined for each cup. For the shoot length, for each cup, fully developed tillers were measured starting from above the basaltic rock until the longest point of each tiller. A similar method was applied to count and measure every spike and auricle lengths within one cup. All shoots from each cup were collected to determine plant fresh weight, and seeds were counted and weighed for each cup. For the spike number and seed number per spike, averages were made based on the amount of plants, spikes and seed number for each cup. Spectrophotometric analyses to determine total flavonoid and polyphenol concentration, as well as pigment concentrations, were conducted on wheatgrass. The determination of (in)soluble sugar content was conducted in wheat grains. Protocols are described in subchapter 1 of the **Supplementary Information**.

### 2.8 Statistical analysis

Outliers were determined using the Grubbs’ test and were excluded from the statistical analysis. For the plant phenotypic screening in function of comparing the responses to different biochars resulting from different pyrolysis parameters, a two-way ANOVA was used. For every other described plant phenotypic parameter, as well as the spectrophotometric measurements, data were analyzed using a one-way ANOVA. Normal distribution and homoscedasticity of data were verified using the Shapiro-Wilk test and Bartlett test respectively. When assumptions were not met, the data were transformed (square root, inverse, exponential or logarithmic). Two-by-two comparisons of all exposure conditions were done using the parametric Tukey’s HSD test. In case normality or homoscedasticity were not met after transformation of data, a non-parametric Kruskal-Wallis test was conducted, followed by the Wilcoxon rank sum test in function of a two-by-two comparison. Differences between conditions were considered significant at the level p < 0.05.

## 3 Results

### 3.1 Determination of pyrolysis temperature: feedstock analysis and biochar yield

**Figure 2:**
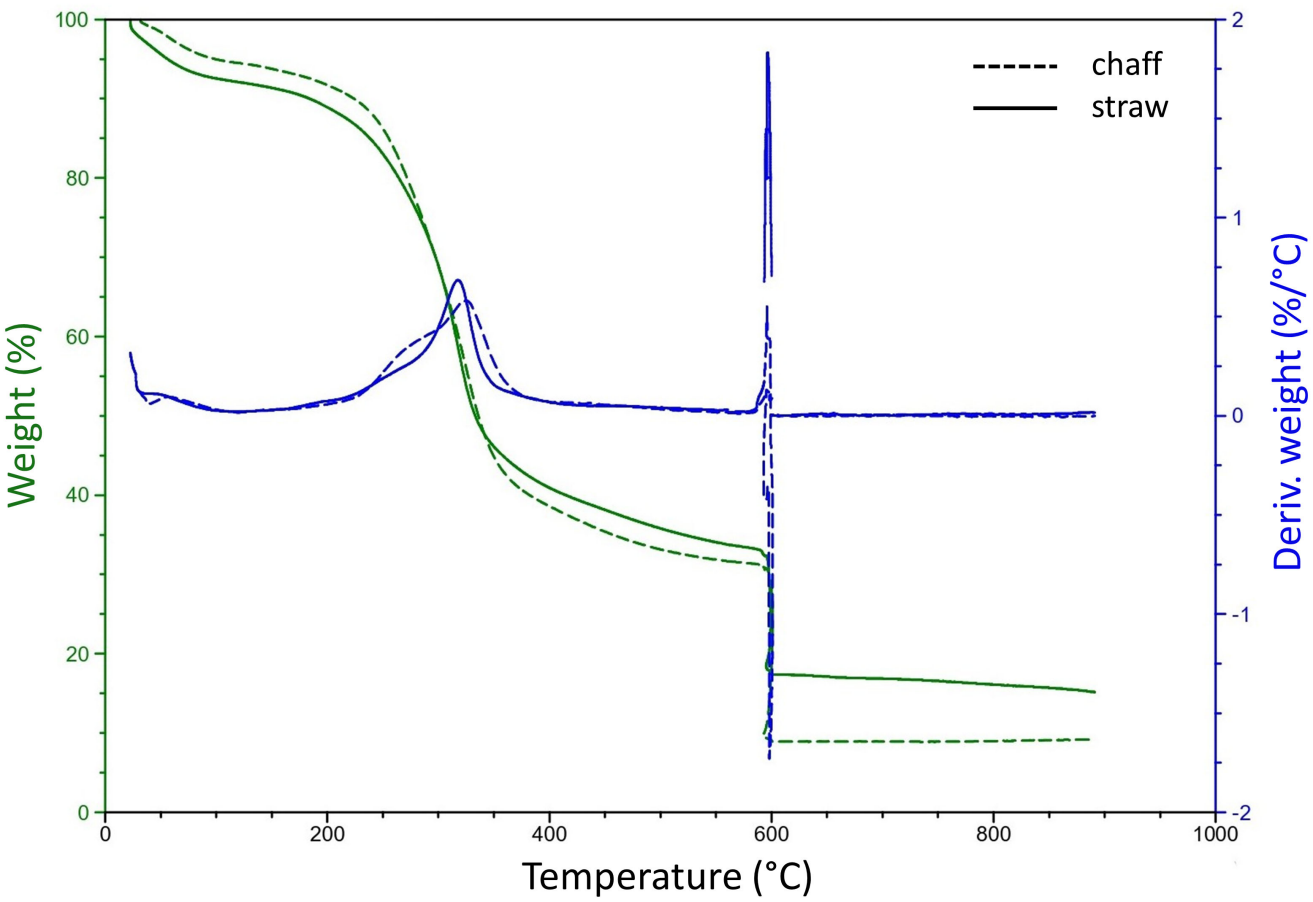
A thermogravimetric analysis of wheat chaff and straw. Dashed and full lines indicate the degradation profiles of chaff and straw respectively. The left (green) y-axis represents the weight percentage of the initial sample weight, the right (blue) y-axis represents a derivative weight loss in function of an increasing temperature (°C).

To determine a suiting pyrolysis temperature for residual materials of wheat crops, a thermogravimetric analysis (TGA) was conducted to determine the degradational profiles, or loss of physical mass due to increasing temperatures, of chaff and straw (**Figure 2**). For both feedstock components, the end of the temperature peak causing the large part of the thermal degradation was determined around 360°C. Further degradation of volatile matter at increasing temperatures also resulted in similar patterns for chaff and straw, only resulting in differing ash contents (profile beyond 600°C, quantified in **Supplementary Table 1**). Two candidate pyrolysis temperatures of 450°C and 600°C were selected to ensure mass degradation past 360°C and to investigate the effect of biochar resulting from different pyrolysis temperature on plant growth. At 450°C, an additional 7% - 8% of volatile matter was degraded in chaff and straw respectively compared to mass degraded at 360°C. At 600°C this was 12%-14% more. Using the small-batch pyrolysis reactor, biochar was produced from wheat straw at 450°C (SBC450, yield: 32%) and 600°C (SBC600, yield: 29%). Using the lab-scale pyrolysis reactor, biochar was produced at 450°C using both chaff and straw (WBC450 yield of 30%). **Supplementary Table 2** includes the yields from the produced biochars. Precise temperatures considering the peak of degradation, as well as their corresponding mass loss percentages compared to mass lost at 450°C and 600°C for chaff and straw are indicated in **Supplementary Table 1**.

### 3.2 Plant phenotypic screening of different biochars

**Figure 3:**
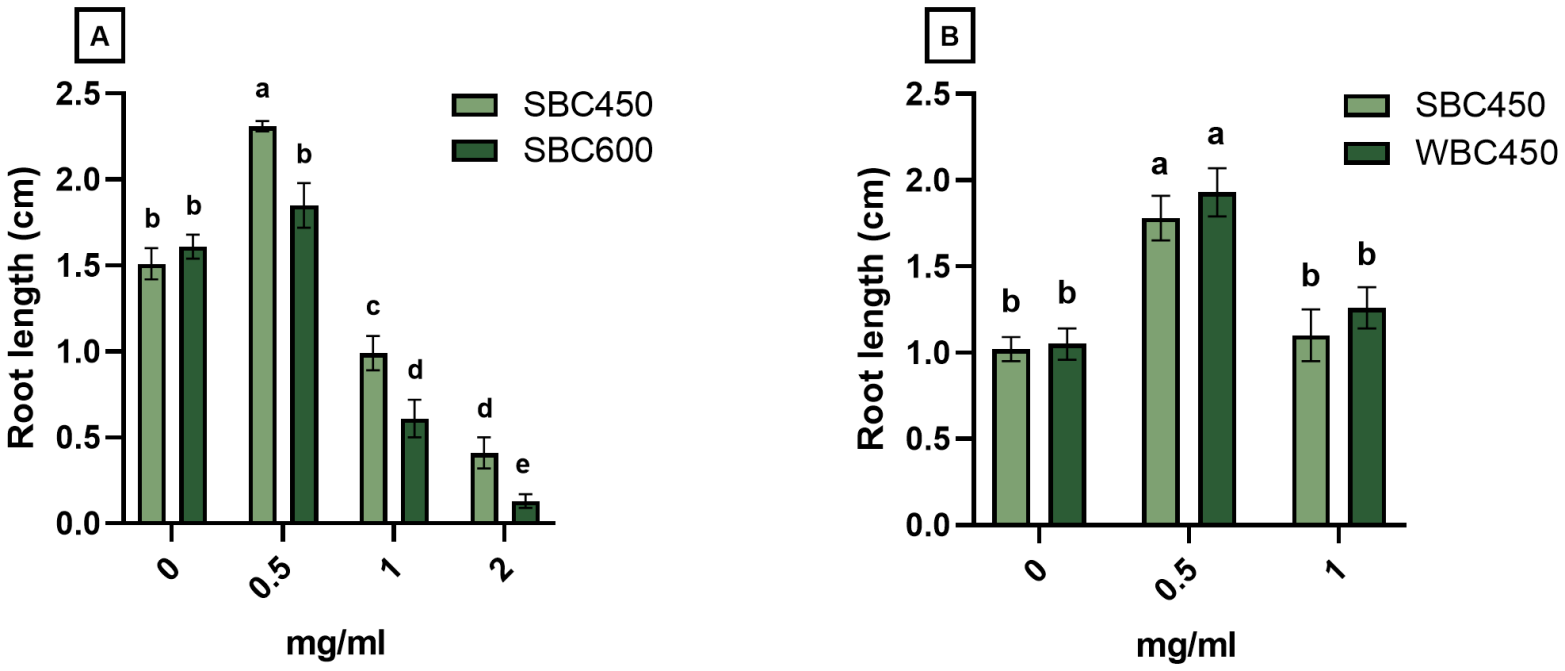
The effect of biochar on root length (cm) of *A. thaliana* seedlings at 7 DAS. Plants were cultivated in a 96-well plate with ¼ MS medium amended with biochar concentrations ranging from 0 mg/ml to 2 mg/ml. **(A)** Plant root lengths of seedlings exposed to either SBC450 or SBC600. **(B)** Plant root lengths of seedlings exposed to either SBC450 or WBC450. Bar plots represent the average ± S.E. of at least 7 biological replicates. Different letters indicate significant differences between conditions (p-value < 0.05, two-way ANOVA).

Produced biochars, resulting from different pyrolysis parameters (temperature, feedstock composition), were compared using a quick plant phenotypic screening using *A. thaliana*. Plant root length was determined at 7 DAS (**Figure 3**), whilst root length and seedling fresh weight were determined at 10 or 11 DAS (**Supplementary Figures 1, 2**).

In the first screening, the plant growth-inducing or -inhibiting effects of SBC450 and SBC600 were compared, to determine the effect of pyrolysis temperature on eventual plant development (**Figure 3A**). At 7 DAS, seedlings exposed to all biochar concentrations had significantly longer roots when exposed to SBC450 compared to SBC600. Notably, only the lowest SBC450 concentration used (0.5 mg/ml) induced a root growth-promoting response compared to the control. When exposed to higher concentrations of biochar (1 mg/ml and 2 mg/ml), root lengths of seedlings exposed to SBC450 and SBC600 significantly decreased compared to plants grown in medium without biochar (**Figure 3A**). The root length of seedlings at 10 DAS (**Supplementary Figure 1A**) displayed similar concentration-dependent responses to biochar. Plant fresh weights were however only significantly higher for plant exposed to 0.5 mg/ml SBC600. Yet, when exposed to the highest concentration of 2 mg/ml SBC600, seedling fresh weights dropped significantly compared to 2 mg/ml SBC450, as well as plants not treated with biochar (**Supplementary Figure 1B**).

To determine the effects of feedstock composition during pyrolysis on eventual plant development after biochar exposure, a similar screening was conducted where SBC450 and WBC450 were compared. Here, no differences on the induced effect of biochar on root lengths of 7-day-old seedlings were observed (**Figure 3B**). However, the concentration-dependent response of seedlings on biochar was evident, where 0.5 mg/ml biochar caused root lengths to increase, while 1 mg/ml biochar did not induce this significant increase. At 11 DAS (**Supplementary Figure 2**), similar patterns were observed, where both biochars significantly increased the root length and fresh weight of exposed seedlings, without observable differences between plants exposed to SBC450 and WBC450.

### 3.3 Biochar (WBC450) characterization and mineral leaching

To determine the effect of biochar on the mineral concentrations of the growing medium used for *A. thaliana* cultivation, a static leaching experiment was conducted using a range of biochar concentrations. These biochar concentrations were similar to the ones used in the screening experiments, with an additional lower concentration of 0.25 mg/ml, as this was of interest for further plant experiments. Resulting pH, electrical conductivity (EC) and mineral concentrations are described in **Table 1**, copper (Cu) concentrations were below the detection limit for all measured samples (0.050 ppm).

**Table 1:** pH, EC (µS/cm) and mineral concentrations (mg/l) in ¼ MS medium after static leaching experiment of 7 days with WBC450.

		*pH*	*EC (µS/cm)*	mineral concentration in ¼ MS medium (mg/l)								
				Ca	K	Mg	P	S	Fe	Mn	Na	Zn
**0 mg/ml**	**av.**	**4.96**	**1359**	**30.572**	**119.399**	**8.803**	**9.871**	**12.374**	**1.268**	**1.369**	**2.495**	**0.427**
WBC450	S.D.	0.04	14	0.067	0.542	0.018	0.072	0.110	0.004	0.004	0.033	0.005
**0.25 mg/ml**	**av.**	**7.10**	**1450**	**31.635**	**137.725**	**11.232**	**11.682**	**13.755**	**1.131**	**1.175**	**2.863**	**0.330**
WBC450	S.D.	0.01	10	0.121	0.029	0.053	0.103	0.125	0.013	0.003	0.015	0.006
*0.5 mg/ml	**av.**	**7.35**	**1520**	**31.120**	**157.052**	**13.759**	**13.633**	**15.298**	**0.949**	**0.972**	**3.384**	**0.306**
WBC450	S.D.	0.18	25	0.025	3.199	0.256	0.159	0.032	0.058	0.024	0.141	0.001
**1 mg/ml**	**av.**	**7.77**	**1681**	**27.984**	**189.601**	**17.351**	**15.785**	**17.969**	**0.434**	**0.847**	**3.918**	**0.269**
WBC450	S.D.	0.14	20	0.653	0.782	0.050	0.123	0.143	0.034	0.012	0.057	0.004

Presented values are average (av.) ± S.D. of 3 replicates. An asterisk indicates conditions where values are average (av.) ± S.D. of 2 replicates.

As the WBC450 concentration amended to the medium increased, both pH and EC rose as well. This trend was also seen in most macronutrients (K, Mg, P, and S) as well as Na, wherein elevated WBC450 concentrations resulted in increased mineral concentrations in the growing medium. Calcium concentrations remained relatively stable, except at the highest WBC450 concentration of 1 mg/ml, where a slight decrease was observed. The remaining micronutrients (Fe, Mn, and Zn) demonstrated an opposing trend, where growing medium mineral concentrations decreased at higher biochar concentrations. **Supplementary Table 2** contains the results of the elemental composition (C, H, N, O and S) of WBC450 biochar, as well as the volatile matter and ash contents.

### 3.4 Biochar influences plant ploidy level

**Figure 4:**
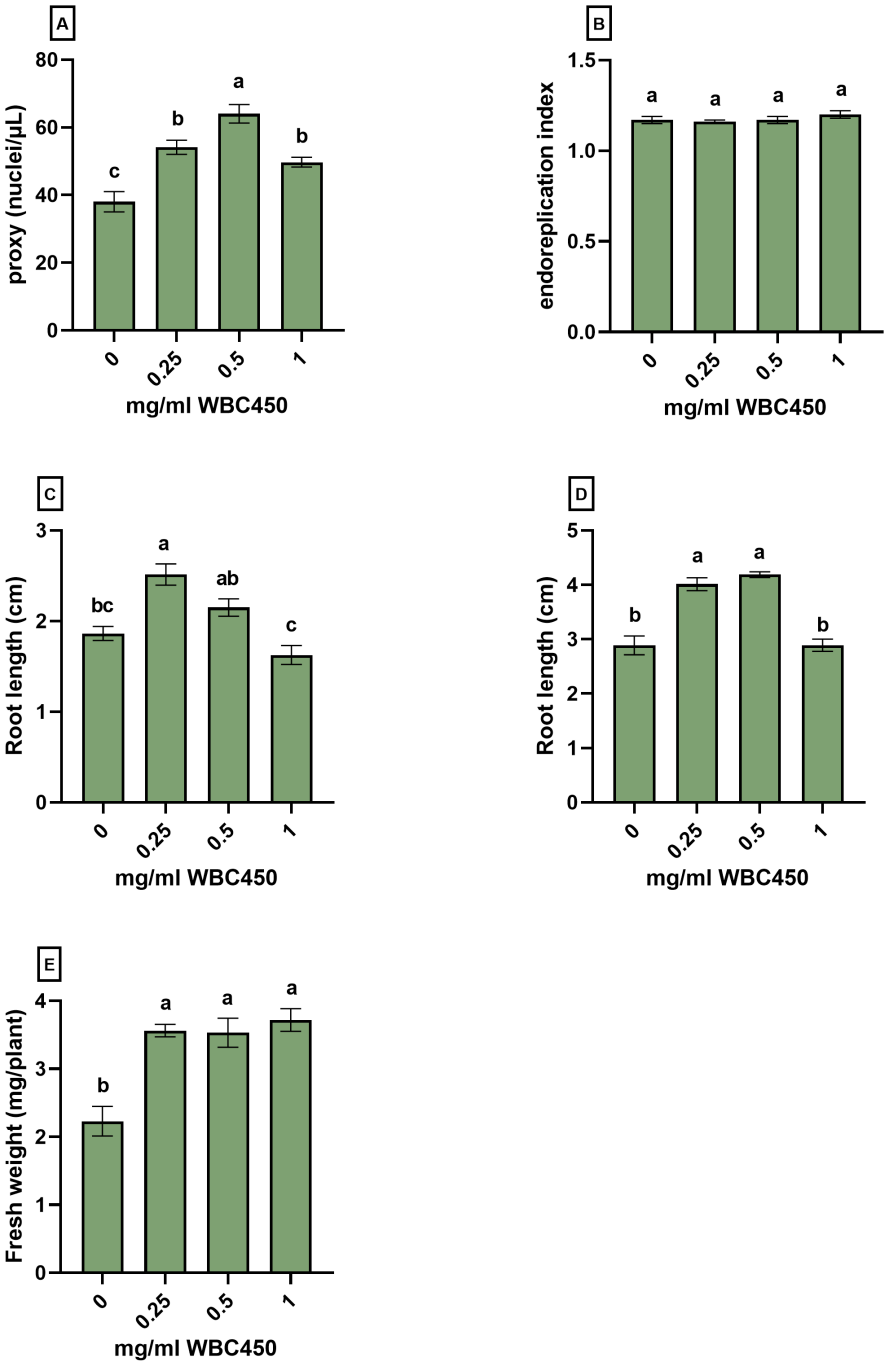
Nuclear ploidy level analysis and phenotypic measurements of *A. thaliana* seedlings exposed to WBC450. Plants were cultivated in a 96-well plate with ¼ MS medium amended with WBC450 biochar concentrations, ranging from 0 mg/ml to 1 mg/ml. At 7 DAS, plants were harvested for nuclear ploidy level analysis, quantifying the **(A)** proxy (nuclei/µL) and the **(B)** endoreplication index, as well as a the **(C)** root length (cm). At 10 DAS, measurements of **(D)** root lengths (cm) and **(E)** fresh weights (mg/plant) were conducted. Bar plots represent the average ± S.E. of at least 7 biological replicates. Different letters indicate significant differences between conditions (p-value < 0.05, one-way ANOVA).

To further elucidate the effect of biochar on plant development, *A. thaliana* seedlings were cultivated in the same way as in the phenotypic screening systems. On 7 DAS, root lengths were measured and samples were taken for flow cytometric analysis of the nuclear ploidy levels. On 10 DAS, root lengths and seedling fresh weights were determined (**Figure 4**).

At 7 DAS, root lengths significantly increased when seedlings were exposed to 0.25 mg/ml WBC450, and an increasing trend was observed at 0.5 mg/ml WBC450. The increase in root length was significant for seedlings exposed to 0.25 mg/ml and 0.5 mg/ml WBC450 at 10 DAS. In addition, plant fresh weights increased significantly when treated with all biochar exposure concentrations (**Figure 4C–E**). At 7 DAS, the proxy (counted nuclei per µL as an estimation of cell division) increased significantly in seedlings exposed to all biochar concentrations, with the highest value at 0.5 mg/ml WBC450. However, when looking at the endoreplication index (EI), no significant differences were observed (**Figure 4A, B**).

### 3.5 Biochar increases yield of wheatgrass

In order to translate the results from biochar exposure experiments on *A. thaliana* to cultivations using wheat, WBC450 was implemented in a hemp-based growing system to investigate the effect of biochar on wheat development. In the initial screening steps, as well as the experiment considering plant ploidy levels, it became apparent that growth-inhibiting or neutral effects of the highest biochar concentrations on plant phenotypic parameters at 7 DAS, became growth-stimulating or remained neutral compared to the controls after a prolonged exposure of 10/11 DAS (**Figure 3**; **Supplementary Figures 1, 2**; **Figure 4C–E**). Therefore, in the hemp-based cultivation of wheat seedlings, an intermediate check on early seedling development was conducted at 7 DAS, complemented with endpoint measurements on 14 DAS.

**Figure 5:**
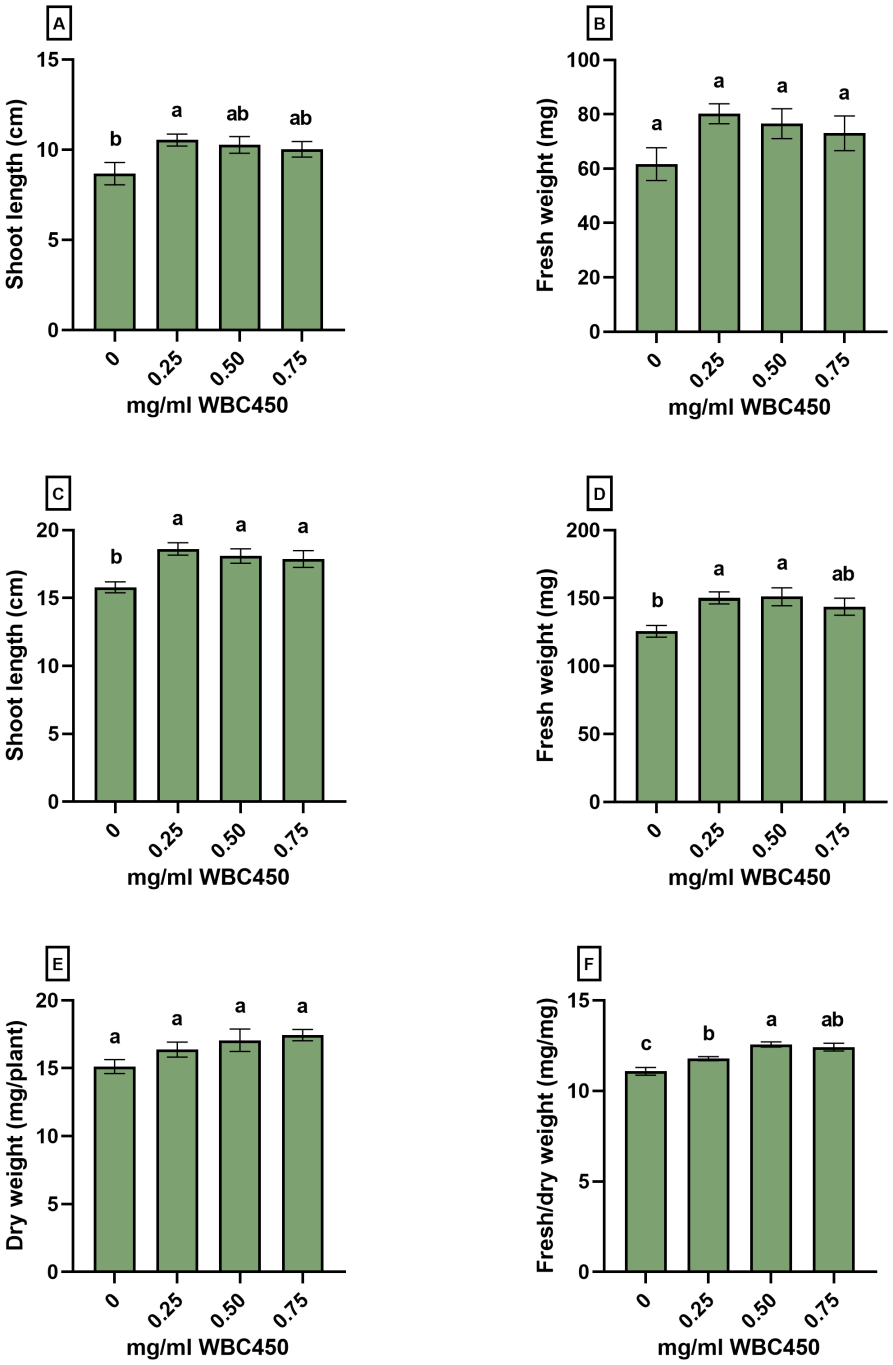
Phenotypic measurements of wheat seedlings exposed to WBC450. Parameters quantified in wheat shoots grown on hemp mats and exposed to WBC450 biochar. At 7 DAS, **(A)** shoot length (cm) and **(B)** shoot fresh weight (mg/plant) were measured. At 14 DAS, **(C)** shoot length and **(D)** fresh weight were measured, as well as the **(E)** dry weight (mg/plant) and a **(F)** shoot fresh/dry weight ratio (mg/mg). Bar plots represent the average ± S.E. of at least 8 biological replicates. Different letters indicate significant differences between conditions (p-value < 0.05, one-way ANOVA).

Amendment of 0.25 mg/ml WBC450 caused a significant increase in shoot length at 7 DAS, and an increasing trend in fresh weight was observed when shoots were exposed to any biochar concentration (**Figures 5A, B**). At 14 DAS, all biochar exposure conditions elicited a significant increase in shoot length (**Figure 5C**), and exposure to 0.25 mg/ml and 0.5 mg/ml WBC450 significantly elevated the fresh weight of wheatgrass compared to the control (**Figure 5D**). No significant differences were observed in the dry weight (**Figure 5E**), however, all biochar concentrations did significantly increase the fresh/dry weight ratio of the wheat shoots (**Figure 5F**).

An estimation of seedling quality was done using spectrophotometric measurements of flavonoid concentration at 7 DAS, repeated on 14 DAS complemented with total antioxidative capacity, malondialdehyde concentration and pigment content (**Supplementary Figures 3, 4**). There were no observable differences between control and WBC450-exposed shoots at 7 DAS (**Supplementary Figure 3**), nor at 14 DAS (**Supplementary Figure 4**). Mineral concentrations in shoot tissues were also quantified at 14 DAS (**Table 2**). Exposure to WBC450 biochar significantly increased the concentrations of K and S. However, shoots of seedlings exposed to the lowest concentration of 0.25 mg/ml had a significantly lower Na concentration, and 0.5 mg/ml biochar induced a significant reduction in Fe concentrations in shoots, compared to the controls.

**Table 2:** Mineral concentrations (mg/g dry weight) in 14-day-old wheat seedlings exposed to WBC450.

		Wheat seedling mineral content (mg/g dry weight)									
		Ca	K	Mg	P	S	Fe	Mn	Na	Zn	Cu
**0 mg/ml**	**av.**	**1.81ab**	**23.55a**	**1.74a**	**6.02a**	**2.35a**	**0.17a**	**0.064ab**	**0.24a**	**0.029a**	**0.0067a**
WBC450	S.E.	0.10	0.93	0.09	0.3	0.09	0.08	0.003	0.02	0.001	0.0004
**0.25 mg/ml**	**av.**	**1.83ab**	**26.22b**	**1.86a**	**6.04a**	**2.62b**	**0.058ab**	**0.060b**	**0.12b**	**0.030a**	**0.0065a**
WBC450	S.E.	0.05	0.30	0.02	0.28	0.03	0.003	0.005	0.003	0.001	0.0003
**0.5 mg/ml**	**av.**	**1.88a**	**26.67b**	**1.87a**	**6.68a**	**2.67b**	**0.051b**	**0.074a**	**0.19a**	**0.032a**	**0.0069a**
WBC450	S.E.	0.06	0.44	0.05	0.22	0.04	0.002	0.003	0.006	0.002	0.0002
**1 mg/ml**	**av.**	**1.58b**	**26.50b**	**1.67a**	**6.03a**	**2.65b**	**0.072ab**	**0.062ab**	**0.18ab**	**0.032a**	**0.0071a**
WBC450	S.E.	0.08	0.48	0.09	0.16	0.05	0.015	0.003	0.025	0.002	0.0004

Mineral concentrations are quantified in wheat shoots grown on hemp mats and exposed to WBC450 biochar. Presented values are average (av.) ± S.E. of at least 4 biological replicates. Different letters next to the average values indicate significant differences between conditions within minerals (p-value < 0.05, one-way ANOVA).

### 3.6 Implementation of biochar in a hydroponic cultivation of wheat

**Figure 6:**
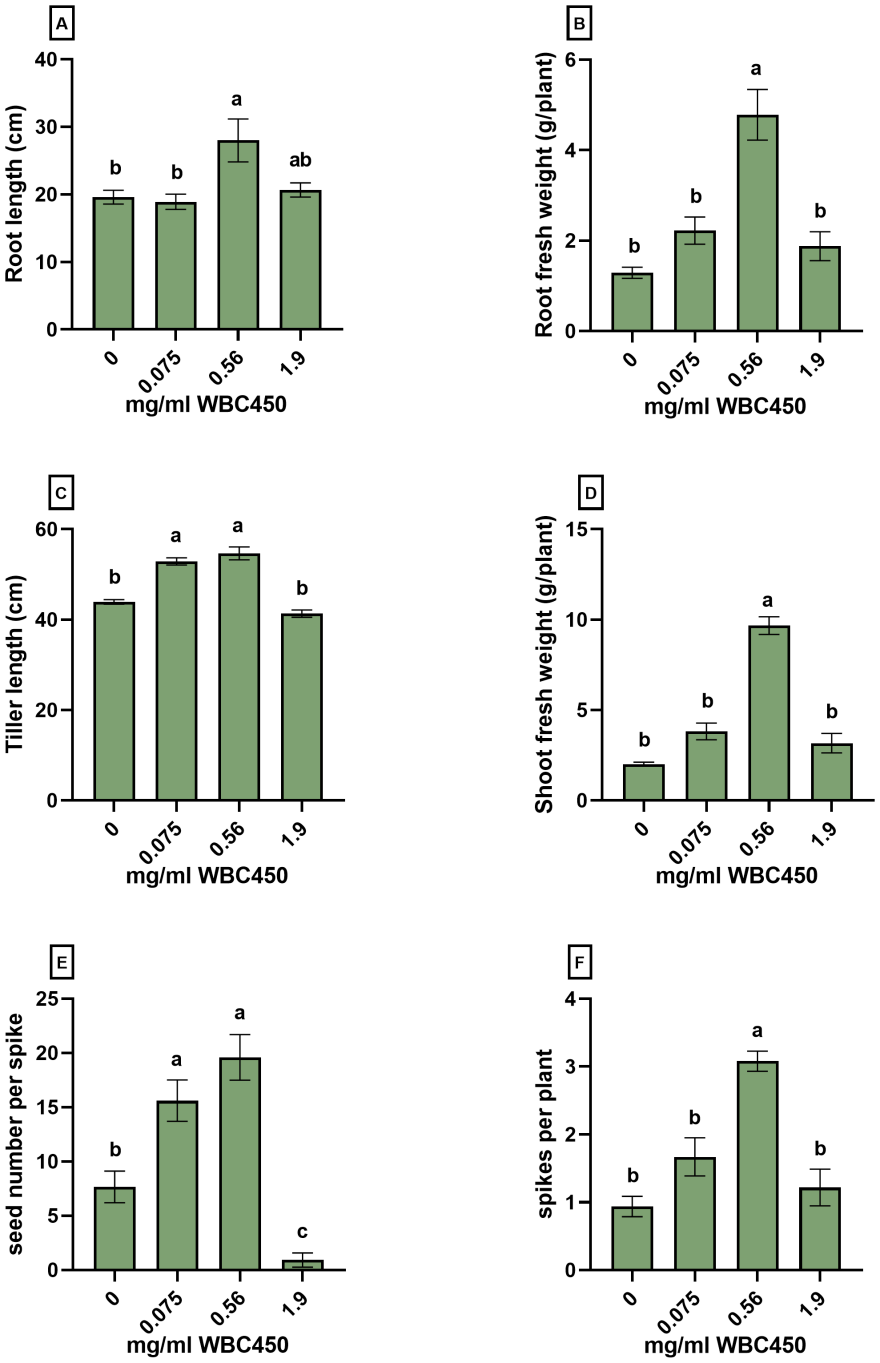
Phenotypic measurements on roots, shoots and seeds of mature wheat exposed to WBC450. Parameters were quantified in mature wheat plants cultivated hydroponically and exposed to WBC450 biochar. At 91 DAS, measurements of the **(A)** root length (cm) and **(B)** root fresh weight (g/plant), the **(C)** average tiller length (cm) and **(D)** shoot fresh weight (g/plant) as well as the **(E)** seed number per spike and **(F)** the spike count per plant were conducted. Bar plots represent the average ± S.E. of 6 separate cups contained in one exposure cultivation box. Different letters indicate significant differences between conditions (p-value < 0.05, one-way ANOVA).

In the final experiment, the effect of wheat-based biochar on the cultivation of mature wheat was explored in a seed-to-seed experiment. Wheat plants were cultivated hydroponically, exposing the cultivation system to WBC450 concentrations of 0 mg/ml, 0.075 mg/ml, 0.56 mg/ml and 1.9 mg/ml. **Supplementary Table 3** contains the pH and EC values of the ½ Hoagland solutions before refreshing. **Figure 6**; **Supplementary Figure 5** display the phenotypic measurements on roots, shoots, spikes and seeds after 96 days of cultivation. Tiller length (**Figure 5C**), and the spike length (**Supplementary Figure 5A**) significantly increased when wheat plants were exposed to the lowest WBC450 concentration of 0.075 mg/ml. The WBC450 concentration of 0.58 mg/ml caused a clear and significantly yield-promoting effect on all measured parameters (**Figure 6**; **Supplementary Figure 5**). For root fresh weights, shoot fresh weights and spike number, this beneficial effect was even significantly higher for plants exposed to 0.58 mg/ml as compared to all other exposure conditions (**Figure 6B, D-F**). In contrast, when plants were exposed to 1.9 mg/ml WBC450, the phenotypic parameters of roots and shoots showed no significant beneficial differences compared to the control plants, with an exception for root dry weight (**Supplementary Figure 5C**). Negative effects on yields were even observed in seed number per spike (**Figure 6E**) and seed fresh weight (**Supplementary Figure 5**) when plants were exposed to 1.9 mg/ml WBC450. Exposure to WBC450 did not affect grain starch and sugar content (**Supplementary Table 4**). However, in this measurement, the effect of the highest biochar concentration (1.9 mg/ml) could not be investigated as grain yields were too low when plants were exposed to this biochar concentration.

## 4 Discussion

In the experiments covered in this manuscript, biochars resulting from differing pyrolysis conditions were screened and compared. Subsequently, the impact of biochar on seedling development and implementation of biochar in a hydroponic seed-to-seed experiment for wheat were investigated.

### 4.1 Rationale for WBC450 selection

#### 4.1.1 Determination of pyrolysis temperatures

Joseph et al. (2021) indicated that the potential beneficial outcome of biochar on plant growth could be dependent on the interplay between feedstock, production parameters and application context (Joseph et al., 2021). The current manuscript is one of the few studies that investigates the application of biochar in soilless and even water-based growing media. For this reason, multiple pyrolysis temperatures were chosen in the initial phase of this manuscript, and their effects on plant growth were compared.

Hemicellulose and cellulose are decomposed between 200°C – 300°C and 300°C – 380°C respectively, whilst lignin is degraded between 200°C and 500°C (Yang et al., 2006). This could explain the peak of degradation, ending around 360°C for both chaff and straw (**Figure 2**; **Supplementary Table 1**). A more stable continuation of degradation after this peak was observed, explained by the further degradation of lignin, as well as the release of more volatile matter with increasing temperatures (He et al., 2018; Lataf et al., 2022). When reducing pyrolysis temperatures below the needed temperature to degrade hemicellulose, the feedstock would only generate small amounts of tar and gas (He et al., 2018). Therefore, the lowest pyrolysis temperatures used in this manuscript was chosen well beyond the degradational peak, at 450°C.

Lataf and colleagues investigated and compared the agronomic suitability of biochars produced at from different feedstock materials at different temperatures. They noticed that an increasing pyrolysis temperature can increase the ash content and inorganic carbon, potentially influencing EC and pH as will be discussed below. As the degradation of volatile matter, and so the probable increase in ash content, was higher at 600°C as compared to 450°C, potentially producing a biochar with different physicochemical characteristics, 600°C was chosen as the second pyrolysis temperature.

#### 4.1.2 Plant-based screening of biochars

Overall, it became evident from the comparison between different biochars on plant growth parameters that SBC450 more often induced stronger growth-promoting responses in the *A. thaliana* seedlings (**Figure 3A**; **Supplementary Figure 1**). Because the results of the biological screening, combined with the fact that producing biochars at lower temperatures reduces production costs, SBC450 was considered for the next comparative experiment. In a second comparison, biochar produced from wheat straw alone (SBC450) was compared with biochar produced from the more complete feedstock, namely wheat straw combined with chaff (WBC450). The degradational profiles of wheat straw and chaff were very similar (**Figure 2**), and the comparison of these biochars did not yield any differences when considering the biological screening (**Figure 3B**; **Supplementary Figure 2**).

As WBC450 is produced at the most energy-efficient pyrolysis temperature tested, consisted of the most complete feedstock for our experimental set-up. This is beneficial in the perspective of circular economy, and WBC450 was therefore chosen for more in-depth characterization and research into its potential in wheat cultivation systems.

### 4.2 The effect of biochar on cultivation conditions

A review by Wang et al. (2020) summarized studies determining the ash content of different straw-based (e.g. wheat, rice, corn) feedstocks and biochars. In this overview, ash content in wheat straw ranged from 2% to 12.97%, and biochars produced from wheat straw were reported to have ash levels of 17.61% at a production temperature of 350°C to ash levels of 40.08% in biochar produced at 700°C (Wang et al., 2020). The ash concentration determined in WBC450 is relatively high (35.8%, **Supplementary Table 2**), which could be explained by the high ash contents found in the feedstock material. The observation of high ash contents in the feedstock material in their turn can be attributed to the fact that it came from previous hydroponic cultivations, which might cause an inherently increased mineral content of the plant material. This was also observed in stems of strawberry plants, where Jeon et al. (2019) found a near 10-fold increase in total mineral content in strawberry (*Fragaria × ananassa* Duchesne) stems cultivated hydroponically (17.7 g/kg), compared to those cultivated in soil (1.8 g/kg) (Jeon et al., 2019).

Biochars with high ash contents are researched for their potential capacity to supply nutrient elements to the growing media they are amended to (Smider and Singh, 2014). Therefore, the effect of WBC450 on the ¼ MS medium, used for the cultivation of *A. thaliana*, was investigated using a static leaching experiment. Biochar amendment to the medium increased the pH and EC and the concentration of most macronutrients, whilst lowering the concentration of most micronutrients (**Table 1**). The EC value can be used as an estimation of the total electrolyte concentration in a solution (Ding et al., 2018), potentially influenced by biochar amendment. In the hydroponic growing system, EC values (**Supplementary Table 3**) of the ½ Hoagland solution amended with WBC450 were also consistently higher up until 70 DAS. Stabilization after 70 DAS could be due to the re-use of the same batch of biochar (in dialysis bags) throughout the whole experiment. Increasing EC-values and mineral content in growing media due to biochar has been observed and investigated by other researchers. In a study investigating the effect of wheat-based biochar amendment to soil, biochar increased the P and K concentrations of soil leachate (Huang et al., 2021). In addition, Vercruysse et al. (2024) observed in their study, investigating common ivy (*Hedera helix* L.)-derived biochar, that biochar amendment to the growing medium also increased its pH and EC. Additionally, a strong correlation between the EC and the K concentration of the biochar-amended medium was observed (Vercruysse et al., 2024). Thus, in current study, the increase of EC can be due to higher potassium concentrations, elicited by increasing biochar concentrations in the growing medium. Lataf et al. (2022) investigated the correlation between EC and the total mineral content of a wide variety of biochars, as well as the role of water-available concentration of these biochar-derived minerals. The researchers found that the total K concentration of biochar was sufficient to model EC with an R^2^ of 0.92, additionally to moderate correlations of water-available K with EC (Lataf et al., 2022). Blok et al. (2017) compared wood-based and vegetable waste-based biochars for peat replacement in soilless plant cultivations. The latter had higher EC and pH-values, related to a higher ash content. Results from this study led the researchers to conclude that nutrient-rich biochars might not be optimal as a direct peat replacement in commercial setups (Blok et al., 2017). An increase in EC and pH resulting from biochar application has its concentration-dependent effect on plant growth, which will be discussed below. For future research, it could be interesting to continuously monitor the EC, integrated in the plant cultivation system, as this can give information on the plant water use efficiency. An increase in EC over time indicates that water is taken up faster than nutrients, whilst a decreasing EC-value would indicate the opposite (Langenfeld et al., 2022). This potentially explains the stabilizing or reducing effect of biochar on the EC in the hydroponic cultivation system after 70 DAS (**Supplementary Table 3**).

While recognizing the high ash content of WBC450, resulting in higher EC and macronutrient concentrations, we aimed to assess its viability in cultivation systems such as hemp substrates and hydroponics. Using low amounts of biochar in the cultivation systems across this study, prevented the use of mitigation strategies, such as washing biochar (Vaughn et al., 2015), to reduce potential negative effects of too high EC values of biochar-amended growing media. The sparing use of biochar in current cultivations leaves biochar left-overs for a variety of other applications, which can be an economically interesting choice. For further research into biochar applications in soilless cultivation systems, special attention should be drawn to the possible mitigating or aggravating effects of biochar on the accumulation of (saline) ions in substrates or recirculating nutrient solution, which is already one of the main difficulties in the fertigation of these systems (Venezia et al., 2022).

### 4.3 The concentration-dependent effect of WBC450 on plant growth

Biochar exposure elicited beneficial effects on growth across all cultivation systems, encompassing the 96-well cultivation of *A. thaliana*, the hemp substrate-based cultivation of wheatgrass, and in the seed-to-seed experiment of hydroponically cultivated wheat. This contributes to the overall beneficial effect of biochar on plant growth in substrate-based cultivations (in 77.3% of studies at specific exposure concentrations) as reviewed by Huang and Gu (2019) (Huang and Gu, 2019).

During the first screening experiments of different biochars in the 96-well plate cultivation system, the biochar concentration of 0.5 mg/ml WBC450 always elicited the highest values when looking at the root lengths and fresh weights of *A. thaliana* seedlings (**Figure 3**, **Supplementary Figures 1, 2**). Therefore, a lower concentration of 0.25 mg/ml was considered in the experimental set-up for the flow cytometric analysis, and the hemp-based cultivation of wheatgrass. Here, we observed that 0.25 mg/ml WBC450 already significantly increased the root length and fresh weight of *A. thaliana* seedlings (**Figure 4C–E**), as well as shoot lengths, fresh weight and fresh/dry weight ratio of wheatgrass (**Figure 5A, C, D, F**). Conclusively, whenever a phenotypic parameter displayed significant beneficial effects induced by biochar, it was always already visible at the lowest concentration used. When plants were exposed to higher concentrations, the beneficial effect of biochar on these phenotypic parameters either stagnated, or values became significantly lower than those observed after exposure to the lowest biochar concentration or even the control condition. Noticeably, trends in results for higher exposure conditions can differ between phenotypic parameters within one experiment. For example, the fresh weights of *A. thaliana* seedlings exposed to 1 mg/ml WBC450 was significantly higher at 10 DAS, where the root lengths did not differ from the control seedlings (**Figure 4D, E**). Similarly, the fresh/dry weight ratio of 14-day-old wheatgrass is significantly higher when exposed to 0.75 mg/ml, whilst the shoot length and fresh weight did not differ from the controls. These results are reflective of the comment made by French and Iyer-Pascuzzi (2018), who stated that the effects of biochar can be dependent on the plant trait studied (French and Iyer-Pascuzzi, 2018). The authors also discussed the importance of research exploring a wide range of biochar application rates in the study design. For example, Solaiman et al. (2012) observed both increasing and decreasing germination rates and seedling growth of wheat, depending on the biochar concentration applied (Solaiman et al., 2012). Our current study considered these observations in the experimental design. As mentioned, although the effect of higher biochar concentrations on plant growth is also plant-trait dependent, lower concentrations consistently induced beneficial effects whenever significant differences were observed compared to controls. This confirms the importance of including a wide range of exposure concentrations when studying the effects of biochar in plant cultivation systems, and considering lower exposure concentrations when seeing inconsistent beneficial effects between plant traits.

The concentration-dependent influence of biochar affects the mineral concentrations in the nutrient solution, correlated with an increase in EC, as discussed above. An optimal pH and EC are important factors when considering the nutrient solution-based cultivation of plants (Putra and Yuliando, 2015). So, the observed concentration-dependent growth responses of plants observed in our experiments could also be attributed to the physical characteristics of the nutrient solutions. Even when all nutrients are present, an increase in EC could cause detrimental salinity effects, resulting in decreases in yield. A pH range of 5.5 to 6.5 is considered optimal for most crop species grown on hydroponics, whilst values outside 5 and 7 could induce growth restriction. Yet, this can be dependent on the crop studied (Putra and Yuliando, 2015). Additionally, pH levels could influence the solubility of different elements, increasing or reducing their availability (Langenfeld et al., 2022). In conclusion, the interplay between EC and pH could be influential to the observed effect of biochar on plant growth and crop yield.

The increase in plant yield, observed across cultivation systems, can also be the result of the specific increase in macronutrient availability. Wheatgrass contained higher levels of K and S, and lower levels of Na and Fe at specific biochar exposure concentrations. For K, S and Na, this can be explained by higher or lower nutrient availability, as observed in the biochar leaching experiment in the ¼ MS medium (**Table 1**). However, as these were different cultivation systems, caution should be taken to make final conclusions. In a study by Zhang et al. (2014), nutrient availability in the growing medium of *Goeppertia insignis* (W.Bull ex W.E.Marshall) increased when coconut coir-based biochar was applied, resulting in an increased N, P, and K content in leaves (Zhang et al., 2014). Nutrient availability is reviewed as an important factor responsive to application of biochar in both soil (Joseph et al., 2021) and soilless substrates (Huang and Gu, 2019), influencing plant growth. Yet, it should be considered that these effects can be dependent on the feedstock and pyrolysis parameters of biochar production (Amery et al., 2021; Lataf et al., 2022).

### 4.4 The effect of WBC450 on the early development of plants

In their review on the use of biochar from main cereals on plant growth, Martinez-Gomez et al. (2022) highlighted the lack of knowledge on the internal regulation mechanisms in plants exposed to biochar (Martinez-Gomez et al., 2022). Therefore, a flow cytometric analysis of *A. thaliana* seedlings was conducted to gain insights in the effect of biochar on cell division (**Figure 4A**) and the endoreplication (**Figure 4B**). Endoreplication is an alternative cell cycle (endocycle), consisting of only the G_1_ and S phase without mitotic cell division, resulting in endopolyploidy (Gutierrez, 2016). There is an implied role for endoreplication in cell growth, as nuclear ploidy levels often correlate with cell size (Lee et al., 2009). Yet, in *A. thaliana* seedlings, despite having longer roots when exposed to 0.25 mg/ml as compared to the controls (**Figure 4C**), the endoreplication index did not increase. The proxy, an indicator for cell division, did increase significantly in seedlings exposed to all biochar concentrations. This indicates that the growth stimulating effect of biochar can be resulting from more cells, instead of cells with a higher level of endopolyploidy. Yet, this conclusion could be premature, as the extent of endoreplication can strongly depend on the cell type studied (Sugimoto-Shirasu and Roberts, 2003). Vandionant et al. (2023) recently demonstrated that the Cd-induced effect on the endocycle is varying when studying individual leaves of differing ages (Vandionant et al., 2023). In the current study, complete seedlings were used, possibly obscuring the observations of tissue-specific effects of biochar on cell cycle regulation. Investigating this in separate plant tissues could therefore be an interesting track for future research into the biochar-induced effects on plant development.

In a transcriptomic analysis in *A. thaliana*, grown on soil amended with poplar wood chip-based biochar, Viger et al. (2015) proposed a central role for auxin as a driving regulator of biochar-induced plant responses. They observed an upregulation of genes related to plant growth, whilst genes related to plant defence systems were downregulated (Viger et al., 2015). This included an upregulation of genes related to cell expansion and division, corresponding to our observation of increased cell division in seedlings exposed to biochar.

### 4.5 The implementation of WBC450 in hydroponic cultivation of wheat

The sustainable management of non-edible parts of crops is pivotal for the concept of circular bioeconomy (Santolini et al., 2021), and the central purpose of this study. This was realized by repurposing hydroponically cultivated wheat crop waste as biochar and implementing this into another seed-to-seed cultivation experiment.

The concentration-dependent effect of biochar was also clearly observable in the hydroponic cultivation of wheat (**Figure 6**). Biochar not only stimulated the vegetative growth of the plant, as observed across all cultivation systems used in this research, it also positively affected the reproductive stage by influencing the spike count per plant and seed number per spike (**Figure 6E, F**), whilst not influencing seed sugar and starch content (**Supplementary Table 3**). Comparably, in a field experiment, Lv et al. (2022) attributed the biochar-induced increases in rice (*Oryza sativa* L.) grain yield to the appearance of more spikes per panicle (Lv et al., 2022). In contrast, no convincing beneficial effects of biochar on the flowering parameters (sunflower head, seed dry weight) of sunflower (*Heliantus annuus* L.) plants, cultivated in either open air or under elevated CO_2_ levels in a greenhouse. However, the minimum level of biochar application was 3% (wt.%) of the total substrate weight (Wang et al., 2023). As mentioned earlier, research into lower application concentrations of biochar could elicit different results.

Although the WBC450 biochar application to the hydroponic system increased the seed number per spike (19.59 ± 2.1 when exposed to 0.56 mg/ml), the highest achieved number was still relatively low. Across 390 winter wheat genotypes in a soil-based experiment, studied for the effect of breeding process on grain yield distribution, the mean observed number of grains per spike were 51.27 and 61.07 grains per spike for 180 genetic resources and 210 elite varieties respectively (Philipp et al., 2018). Additionally, Järvan et al. (2012) investigated the effect of sulphur fertilization on wheat grain yield in different Estonian soils throughout several years. They reported numbers ranging from 20.4 seeds per spike in soils without fertilisation to 38.0 in fertilized soils (Järvan et al., 2012). Our highest reported value under biochar-exposed conditions comes close to values observed in unfertilized soils in this Estonian study. This indicates, although the hydroponic set-up might not have been ideal under control conditions, that implementing biochar is an interesting concept to be considered when optimizing soilless cultivation systems of wheat in non-traditional forms of farming. Here, future research should consider the concentration-dependent effect of biochar on the physicochemical characteristics of nutrient solutions (as discussed above), balancing biochar with complemented fertilizer application, to ensure a plant growth-stimulating environment.

The scope of the current study was to fit the circular bioeconomy framework by repurposing crop waste within a soilless cultivation system, realized within a closed hydroponic cultivation system of wheat within the SpaceBakery project. The exploration of integrating biochar into the hydroponic cultivation of a staple crop such as wheat has not been explored much, yet for tomatoes (*Solanum lycopersicum* L.), Dunlop et al. (2015) conducted research with a similar experimental framework. Crop green waste of tomatoes was converted to biochar, and amended to a saw dust-based substrate in which new tomato plants were hydroponically cultivated. Rinsing of the biochar-amended substrates was needed to reduce the EC, and the researchers did not find any significant biochar-induced effects on growth, yield or fruit quality. Yet, the study emphasized that the biochar production process can repurpose cultivation waste in terms of replacing volumes of substrate, thereby closing the waste loop (Dunlop et al., 2015). Awad et al. (2017) also explored the idea of using biochar as a potential hydroponic growing substrate. They found an approximate two-fold yield of leafy vegetables when combining rice husk biochar with perlite, compared to plants cultivated on just perlite. Neogi et al. (2022) acknowledges biochar technology as a solution in attaining circular bioeconomy, and discussed its potential in achieving several United Nations Sustainable Development Goals. The authors highlighted the potential of engineered or enhanced biochars, as well as the importance of a life-cycle assessment and cost-benefit analysis in function of optimising biochar production systems. These factors could be important to enforce the role of biochar in a circular bioeconomy in future research (Neogi et al., 2022).

In the current study, WBC450 biochar was selected after a plant growth screening and implemented in several cultivation systems including the hydroponic cultivation of wheat. Low amounts of biochar were used to bypass the need for EC level-mitigation strategies such as washing of biochar. The application of biochar resulted in growth-stimulating responses across all used cultivation systems for *A. thaliana*, wheatgrass and mature wheat. In this way, the current study contributes to new knowledge on the production potential of staple crop yield in soilless cultivation systems within the framework of closing waste loops for a circular bioeconomy.

## Data Availability

The raw data supporting the conclusions of this article will be made available by the authors, without undue reservation.
