# Supplementary material for: From crop left-overs to nutrient resource: growth-stimulating potential of biochar in nutrient solutions for wheat soilless cultivation systems
Source: Front Plant Sci. 2024 Sep 5;15:1414212. doi: 10.3389/fpls.2024.1414212 (PMC11410626; doi:10.3389/fpls.2024.1414212)
Supplement: Supplementary file 1 [file DataSheet1.docx]

# Supplementary material

## 1 Supplementary methodologies

### 1.1 Wheat grass cultivation Plant phenotypic and spectrophotometric analyses

At 7 DAS, shoot length and shoot fresh weight were determined after cutting shoots at the base of the substrate. At 14 days after sowing (14 DAS), shoot length, shoot fresh and dry weight were determined. At both time points, shoots were taken and snap frozen in liquid nitrogen for spectrophotometric determination of total flavonoid content (TFC) and total polyphenol content (TPC). At 14 DAS, additional samples were taken for pigment determination (chlorophyll and total carotenes). Here, 5 cm was taken from the cotyledon, 1 cm removed from the tip of the leaf. These were snap frozen in safe lock tubes with a spatula tip of NaCO_3_ to prevent oxidation. Each sample consisted of leaf fragments of two separate shoots. Before analysis, two iron beads were added to the tubes, and all samples were shredded using Retch I/201217307 MM400.

The same samples were used to determine TFC and TPC. An extraction was conducted on shredded samples, ice cold 80% (v/v) ethanol (EtOH) was added, vortexed until thawed and centrifuged at 14000 rpm for 30 minutes at 4 °C.

Total flavonoid content was determined using a standard curve of quercitin (diluted in 80% EtOH). Every well contained 20 µL of sample or the quercitin standard (0-100 µg/ml), and was supplemented with 200 µL of reaction mix. The reaction mix for TFC contained 60 µL absolute EtOH, 10 µL 10% (w/v) aluminium trichloride hexahydrate and 10 µL 1 M potassium acetate and 120 µL distilled water. After an incubation period of 30 minutes at room temperature, absorbance was determined at 415 nm using the FLUOstar Omega microplate reader (BMG Labtech, Ortenberg, Germany).

Total polyphenol content was determined using a standard curve of gallic acid (diluted in 80% EtOH). Before analysis, extracted samples were ½ diluted with 80% EtOH. Every well contained 10 µL of sample or the gallic acid standard (0-100 µg/ml). Subsequently, 50 µL of 10% (v/v) Folin-Ciocalteu phenol reagent was added and incubated for 5 minutes in darkness at room temperature. After, 40 µL of 7% (w/v) Na_2_CO_3_ was added. Plates were incubated for two hours in the dark at room temperature. Absorbance was measured at 725 nm using the plate reader.

A spectrophotometric quantification of pigments was done after extracting shredded samples with 80% (v/v) acetone. Samples were vortexed and centrifuged for 5 minutes at 6000 rpm at 4 °C. The 80% acetone functioned as a blank, 200 µL of blank or acetone-extracted sample were pipetted in a 96-well plate. Absorbances were determined at wavelengths 663 nm, 646 nm and 470 nm. Pigment concentrations were determined using following formulas: Chlorophyll a (chla, µg/ml) = 12.21 A663 – 2.81 A646, Chlorophyll b (chlb, µg/ml) = 20.31 A646 – 5.03 A663, Total carotenes (µg/ml) = (1000 A470 – 3.27 chla – 104 chlb)/229.

All spectrophotometric measurements were done using at least 2 technical replicates for each blank, standard or biological sample. Values for individual biological samples were determined by calculating the average of technical replicates, and blank-corrected if needed.

### 1.2 Wheat seed-to-seed experiment: biochar in hydroponic cultivation of wheat: Seed starch and soluble sugar contents

Starch and soluble sugar contents in wheat seeds were determined using the anthrone reagent, using an adapted protocol based on Hansen and Møller (1975) (Hansen & Moller, 1975). Dried wheat seeds were manually pulverized, and 10-20 mg dry mass was transferred to a 2 mL Eppendorf tube, and sugars were extracted using 2 times 1 mL of 80% (v/v) EtOH which was transferred to a 15 mL tube. Total volume was set to 5 mL using 80 % EtOH, and samples were centrifuged for 5 min at 953 g (Rotina 420R, Hettich Zentrifugen). The pellet was used for the insoluble sugar analysis, the supernatant was transferred to another 15 mL tube and used for soluble sugar analyses. To the pellet, 5 mL 1.1% (v/v) HCl was added to hydrolyze the remaining starch, and the mixture was homogenized, followed by a 30 min heating in a 100 °C water bath. After, the total volume was set to 10 mL using distilled water. To determine starch and soluble sugar content, standard solutions of starch and glucose with 0; 0.1: 0.2; 0.4; 0.6; 0.8; and 10 mg starch or glucose ml^-1^ were made in 1.1% HCl and 80% EtOH, respectively. Subsequently, 100 µL of sample or standard were pipetted into a 2 mL Eppendorf tube, and put on ice. Next, 500 µL ice-cold anthone reagent (0.5 g anthrone in 250 ml 72% (v/v) sulphuric acid) was added to each tube. Samples were vortexed and then heated for 11 min in a 100 °C water bath. All samples were cooled on ice and homogenized using a vortex. Absorbances of standards and samples were measured at 630 nm in a polypropylene 96-well plate (UV-star microplate, Greiner Bio-One).

## 2 Supplementary tables and figures

**Supplementary table 1: Temperatures at the peak of degradation derived from the thermogravimetric analysis with corresponding percentages of mass loss for wheat chaff and straw.** OT: onset temperature of degradation peak, PT: degradation peak temperature, ET: onset temperature at the end of degradation peak. Values result from a single measurement for each chaff and straw.

|  | **Onset and peak degradation temperatures (°C)** | | |  |
| --- | --- | --- | --- | --- |
|  | OT | PT | ET |  |
| chaff | 223.14 | 324.63 | 364.70 |  |
| straw | 226.84 | 317.62 | 350.76 |  |
|  | **loss of mass (%) in temperature interval** | | | |
|  | 0 °C - 100 °C | 100 °C - ET | 100 °C - 450 °C | 100 °C - 600 °C |
| chaff | 5.07 | 53.04 | 59.52 | 64.96 |
| straw | 7.45 | 46.43 | 54.41 | 60.19 |

**Supplementary table 2: Elemental composition of WBC450, ash content of feedstock and biochar, biochar yield.** Values (on dry basis) represent the mean ± S.D. of at least 3 replicates. The standard deviation of O was calculated based on the error propagation theory. VM: volatile matter, N.A.: not applicable. Sulfur levels were below detection limit. *: one replicate available.

|  | **ash (wt%)** | **Yield (%)** | **H (wt%)** | **N (wt%)** | **C (wt%)** | **O (wt%)** |
| --- | --- | --- | --- | --- | --- | --- |
| WBC450 | 35.8 ± 0.1 | *30.70 | 2.44 ± 0.06 | 2.0 ± 0.0 | 48.29 ± 0.58 | 11.5 ± 0.7 |
| SBC450 | - | 32.05 ± 1.97 | - | - | - | - |
| SBC600 | - | 29.22 ± 2.11 | - | - | - | - |
| Straw | 13.5 ± 0.1 | - | - | - | - | - |
| Chaff | 6.8 ± 0.1 | - | - | - | - | - |

**
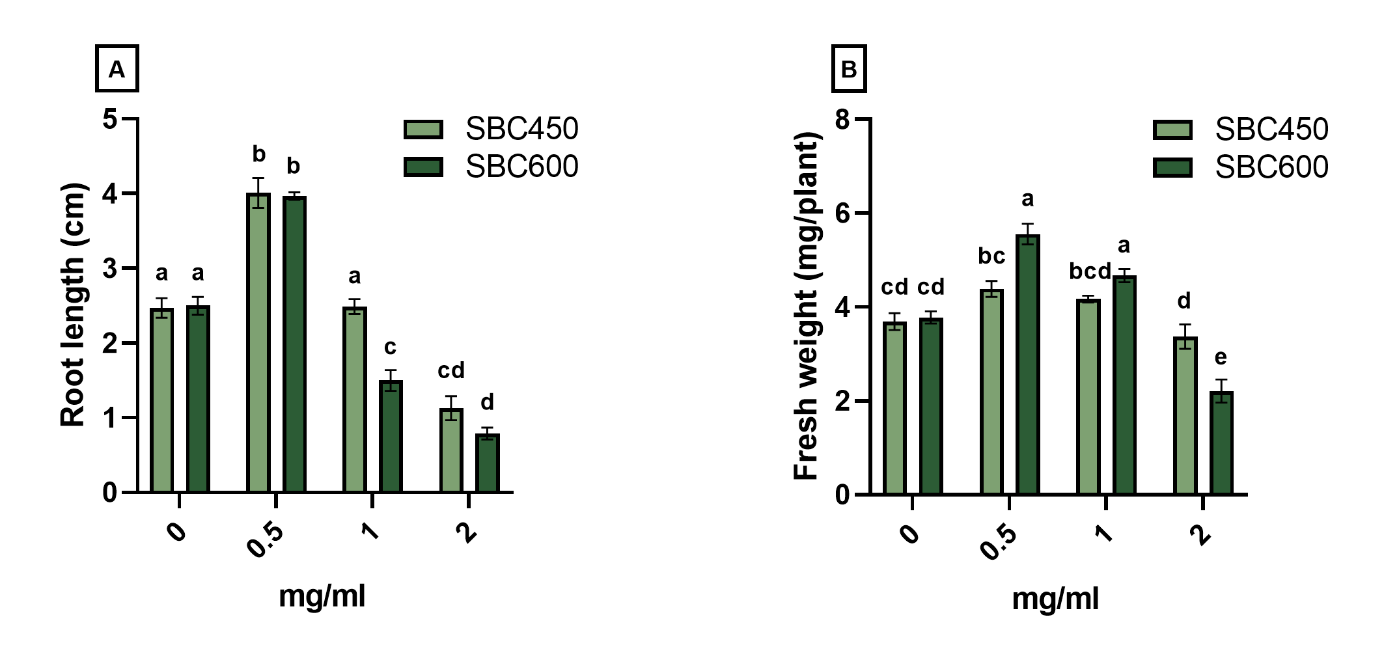
Supplementary Figure 1: Root lengths (cm) and fresh weights (mg/plant) of *A. thaliana* seedlings at 10 DAS.** Plants were cultivated in a 96-well plate with ¼ MS medium amended with biochar concentrations ranging from0 mg/ml to 2 mg/ml. A: plant root lengths of seedlings exposed to either SBC450 or SBC600. B: plant fresh weights of seedlings exposed to either SBC450 or SBC600. Bar plots represent the average ± S.E. of at least 5 biological replicates. Different letters indicate significant differences between conditions (p-value < 0.05, two-way ANOVA).

**
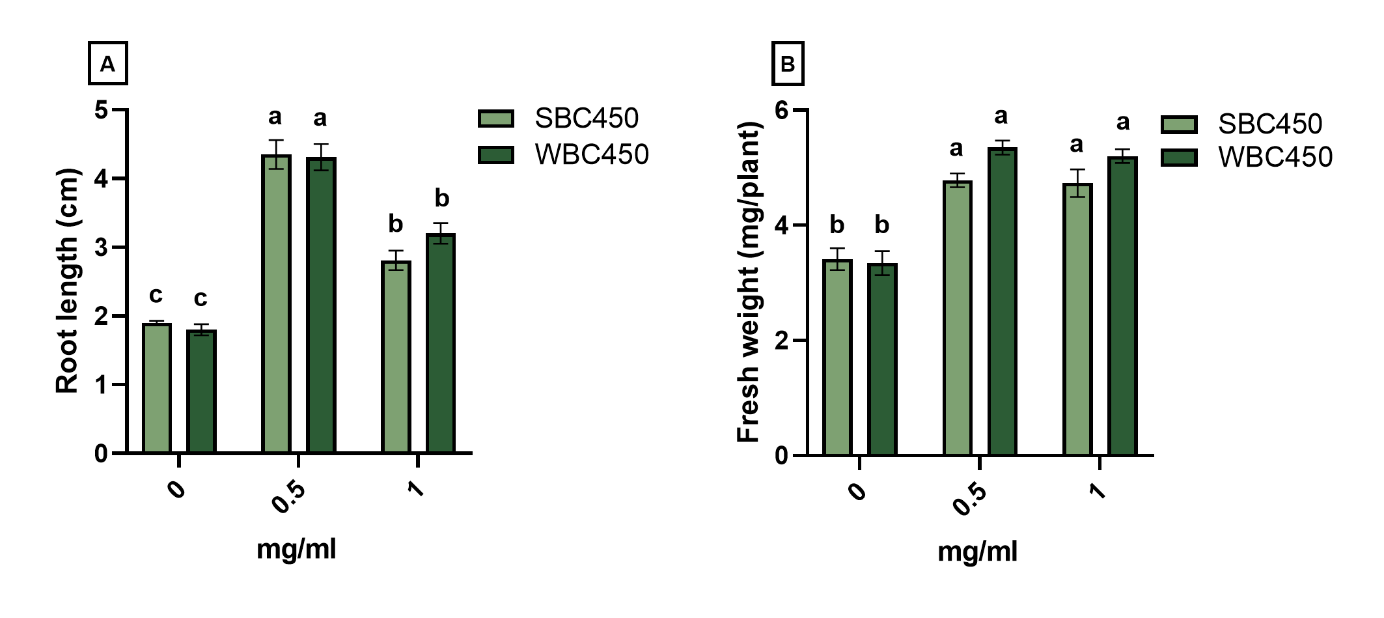
Supplementary Figure 2: Root lengths (cm) and fresh weights (mg/plant) of *A. thaliana* seedlings at 11 DAS.** Plants were cultivated in a 96-well plate with ¼ MS medium amended with biochar concentrations ranging from0 mg/ml to 1 mg/ml. A: plant root lengths of seedlings exposed to either SBC450 or WBC450. B: plant fresh weights of seedlings exposed to either SBC450 or WBC450. Bar plots represent the average ± S.E. of at least 7 biological replicates. Different letters indicate significant differences between conditions (p-value < 0.05, two-way ANOVA).

**
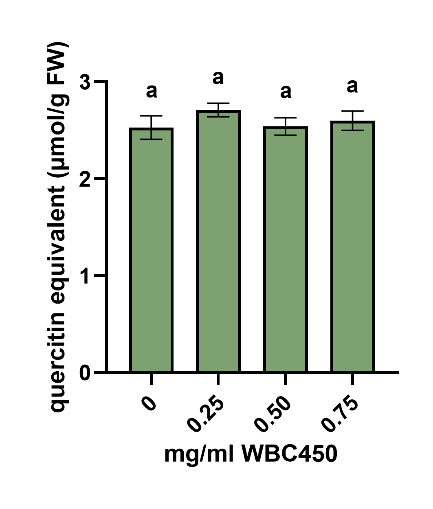
**

**Supplementary figure 3: Total flavonoid content (quercetin equivalent µmol/g fresh weight) in wheat shoots at 7 DAS.** Wheat shoots were grown on hemp mats and exposed to WBC450 biochar. Bar plots represent the average ± S.E. of at least 9 biological replicates. Different letters indicate significant differences between conditions (p-value < 0.05, one-way ANOVA).

**
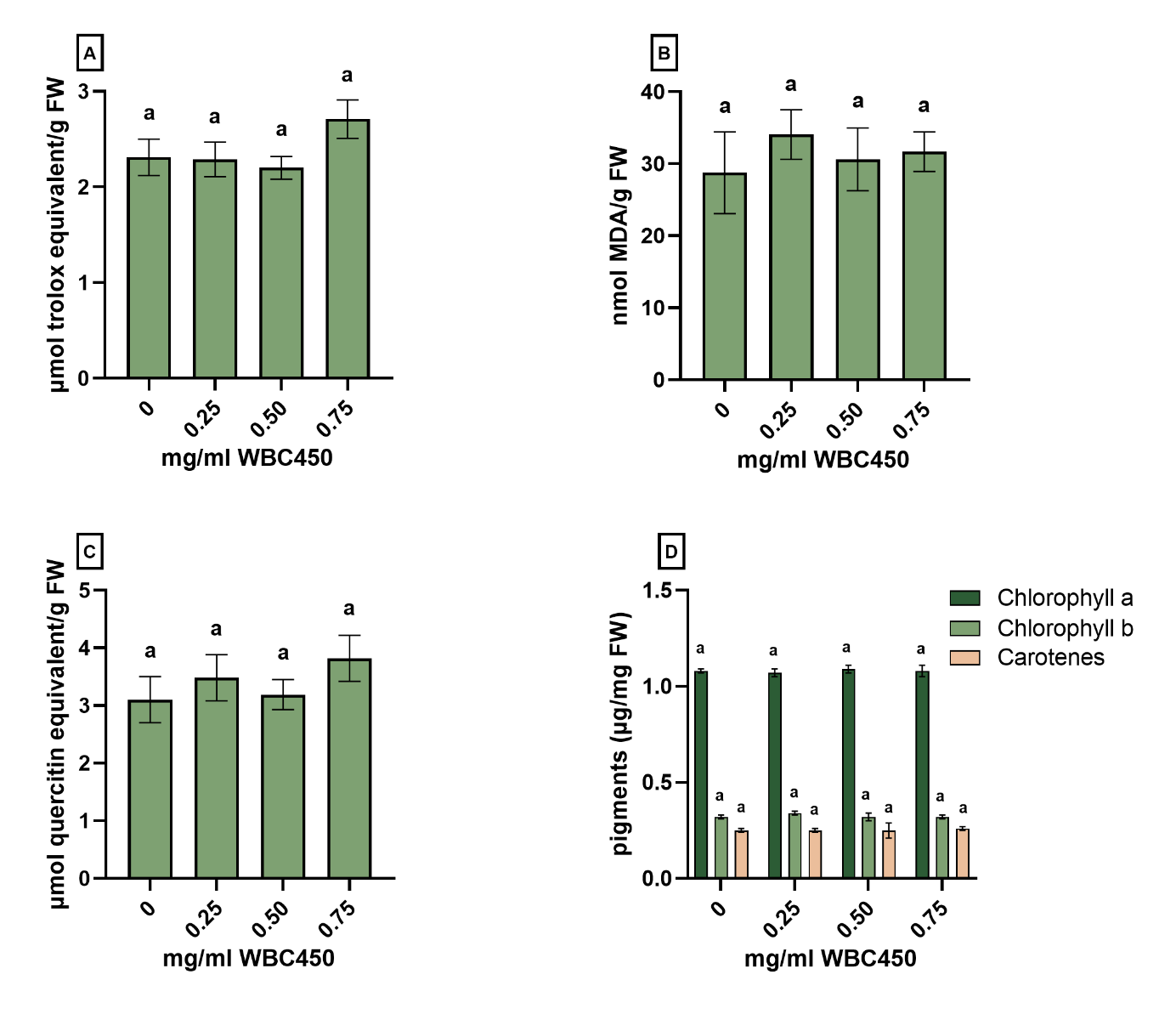
Supplementary figure 4: Biochemical and pigment screening of wheat shoots at 14 DAS.** Parameters quantified in 14-day old wheat shoots grown on hemp mats and exposed to WBC450 biochar. The (A) total antioxidative capacity (µmol Trolox equivalent/g fresh weight), (B) malondialdehyde concentrations as an indicator for lipid peroxidation (MDA/g fresh weight), (C) total flavonoid content (µmol quercetin equivalent/g fresh weight) and (D) pigment concentrations (µg/mg fresh weight) of chlorophyll a, chlorophyll b and total carotenes were determined spectrophotometrically. Bar plots represent the average ± S.E. of at least 6 biological replicates. Different letters indicate significant differences between conditions (p-value < 0.05, one-way ANOVA). Statistical analysis for (D) was done within each pigment type.

**Supplementary table 3: pH and electrical conductivity (µS/cm) of ½ Hoagland solution before refreshing.** The nutrient solution of the hydroponic cultivation of wheat was refreshed every two weeks until the harvest at 91 DAS. Values represent the pH and EC values of one sample of the Hoagland solution.

|  | **14 DAS** | | **28 DAS** | | **42 DAS** | | **56 DAS** | | **70 DAS** | | **84 DAS** | | **91 DAS** | |
| --- | --- | --- | --- | --- | --- | --- | --- | --- | --- | --- | --- | --- | --- | --- |
|  | pH | EC | pH | EC | pH | EC | pH | EC | pH | EC | pH | EC | pH | EC |
| 0 mg/ml | 6.204 | 1283 | 7.135 | 1553 | 6.706 | 1811 | 6.772 | 1631 | 7.191 | 882 | 6.74 | 816 | 6.339 | 1173 |
| 0.075 mg/ml | 6.282 | 1317 | 7.208 | 1529 | 6.98 | 1814 | 7.544 | 1777 | 7.128 | 851 | 7.226 | 802 | 7.055 | 1089 |
| 0.56 mg/ml | 6.933 | 1479 | 7.258 | 1571 | 6.999 | 2490 | 7.193 | 2420 | 7.153 | 1041 | 6.886 | 1045 | 7.235 | 1311 |
| 1.9 mg/ml | 7.511 | 1858 | 7.144 | 1653 | 6.778 | 2160 | 7.323 | 2180 | 7.061 | 1006 | 7.279 | 762 | 7.517 | 982 |

**
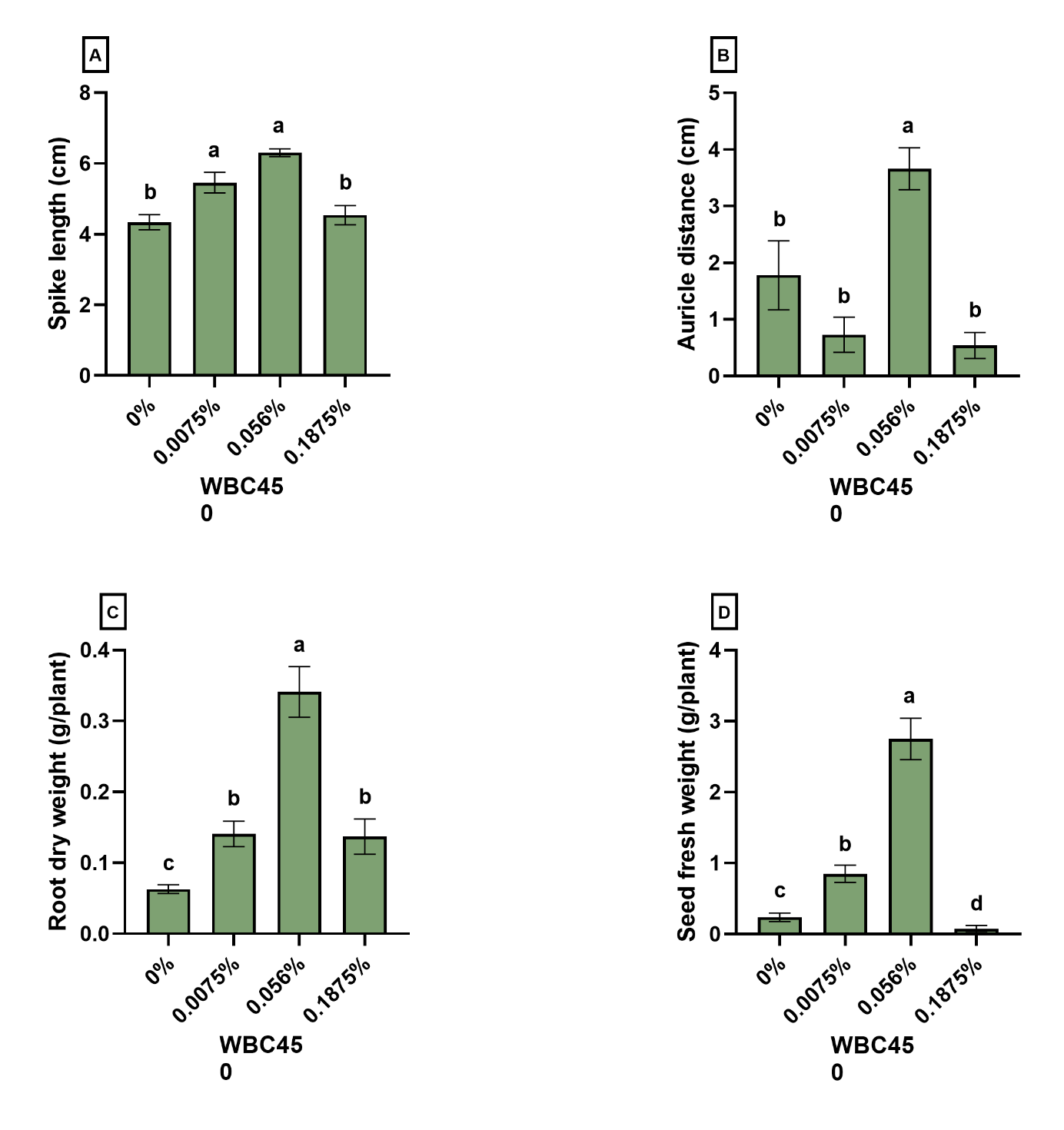
**

**Supplementary figure 5: Average spike and auricle length (cm), root dry weight (g/plant), and seed fresh weight of mature wheat exposed to WBC450 for 91 days.** Parameters were quantified in mature wheat plants cultivated hydroponically and exposed to WBC450 biochar. Bar plots represent the average ± S.E. of 6 separate cups contained in one exposure cultivation box. Different letters indicate significant differences between conditions (p-value < 0.05, one-way ANOVA).

**Supplementary table 4**: **Starch and soluble sugar contents in grains harvested after 91 days of cultivation.** Parameters were quantified in mature wheat plants cultivated hydroponically and exposed to WBC450 biochar. Values represent the averages (expressed as mg sugar or starch per mg grain dry weight (DW)) and an expression in percentage of the total grain dry weight (%) ± S.E. of 5 biological replicates. An asterisk indicates significant differences (p-value < 0.05, one-way ANOVA).

|  | **starch (mg) / DW (mg)** | **% starch in DW** | **sugar (mg) / DW (mg)** | **% sugar in DW** |
| --- | --- | --- | --- | --- |
|  | Average Starch (mg)/ DW (mg) | Average | Average sugar (mg)/ DW (mg) | Average |
| 0 mg/ml | **0.587** ± 0.041 | **58.691** ± 4.092 | **0.022 ± 0.004** | **2.155 ± 0.354** |
| 0.075 mg/ml | **0.566** ± 0.013 | **56.648** ± 1.263 | **0.027 ± 0.002** | **2.654 ± 0.232** |
| 0.56 mg/ml | **0.605** ± 0.019 | **60.525** ± 1.903 | **0.023 ± 0.002** | **2.292 ± 0.243** |
